# The impact of Hepatitis C virus infection on kidney transplantation outcomes: A systematic review of 18 observational studies: The impact of HCV on renal transplantation

**Published:** 2011-04-01

**Authors:** Zohreh Rostami, Mohammad Hossien Nourbala, Seyed Moayed Alavian, Fatemeh Bieraghdar, Yunes Jahani, Behzad Einollahi

**Affiliations:** 1 Nephrology and Urology Research Center, Baqiyatallah University of Medical Sciences, Tehran, IR Iran; 2 Baqiyatallah Research Center for Gastroenterology and Liver Disease, Baqiyatallah University of Medical Sciences, Tehran, IR Iran

**Keywords:** Hepatitis C infection, Kidney transplantation, Graft survival, Patient survival, Mortality, Natural history, Outcome assessment

## Abstract

**Background:**

Hepatitis C virus (HCV) infection occursin 0% to 51% of dialysis patients, and manyHCV-positive patients are urged to undergo kidney transplantation. However, the outcome of renal transplantation in HCV-positive recipients is unknown.

**Objectives:**

Our review aimed to address the outcomesof renal transplantation recipients (RTRs)following kidney transplantation.

**Materials and Methods:**

We selected studies that used the adjusted relative risk (aRR) and 95% CI of all-cause mortality and graft loss in HCV-positive compared with HCV-negative RTRs as study endpoints. Cox proportional hazard analysis was usedin all studies to calculate the independent effects of HCV infection on RTR outcomes. Sixteen retrospective cohort studies and 2 clinical trials were selected for our review. Sixteen studies were related to patient survival, and 12 examined graft survival.

**Results:**

The combined hazard ratio in HCV-infected recipients was 1.69-fold (1.33-1.97, p < 0.0001) and 1.56 times (1.22-2.004, p < 0.0001) greaterthan that of HCV-negative recipients for mortality and graft loss, respectively.

**Conclusions:**

Although HCV-infected RTRs have worseoutcomes than HCV-negative RTRs,kidney transplantation is the preferred treatment for patients with HCV infection and end-stage renal disease.

## Background

Hepatitis C virus (HCV) infection is a common problem among dialysis patients and kidney transplant recipients [1]. The Centers for Disease Control and Prevention (CDC)detects HCV infection by enzyme linked immunosorbent assay (ELISA) in 8.1% (range 0% to 51%) of ESRD patients in large dialysis centers [2]. Additionally, manyHCV-positivepatients are urged to undergokidney transplantation [3]. The major cause of mortality due to liver failure in kidney transplant recipients is HCV infection [4]. The outcome of renal transplantation in HCV-positive recipients is unknown [2][5]; some studies havereported better survival in HCV-positive ESRD patients compared with those remaining on dialysis [1][4][6][7].

A risein viral load following immunosuppression in HCV-positive kidney transplant recipients was suggested to be a significant cause of pooroutcome [1][4][6][8].Also, vifral load and liver deterioration are related [8]. Conversely, several surveys did not observe worse outcomes in HCV-positive renal transplant recipients (RTRs) when HCV infection was acquired before kidney transplantation, especially during the first 5-8 years [7].

However, a recent study from a US registry evaluated the effect of immunosuppressive regimens on survival in HCV-positive RTRs, demonstrating that antibody induction doesnot adversely affect patient survival [1][7][9]. Moreover, cyclosporine [10] and my cophenolat mofetil (MMF) may have protective effects [1][6] and inhibit HCV replication in renal transplant patients with HCV infection. Whether hepatitis virus infected-patients should stay on dialysis or be referred for kidney transplantation remains unknown.

## Objectives

We performed a meta-analysis to determine the effects of HCV infection on outcomes in RT patients.

## Materials and Methods

### Search strategy

We searched electronic databases, including PubMed, the Cochrane Database of Systematic Reviews, EMBASE, and CINHAL, for studies from Jan 1981 to Jan 2010 to identify those that reported the effect of HCV infection on RTR outcomes. Our keywords included "hepatitis C," "HCV infection," "kidney transplantation," "graft survival," "patients survival," "mortality," "natural history," "outcome," and their synonyms. Two authors independently developed a search strategy to identify randomized trials and cohort studies that investigated the effect of HCV on patients and graft survival after kidney transplantation. To identify additional relevant articles, reference lists from qualitative topic reviews and the identified articles were also searched. Duplicate publications were discarded. We restricted our search to human studies and placed no restrictions on language.

### Study Selection

**Figure 1:**
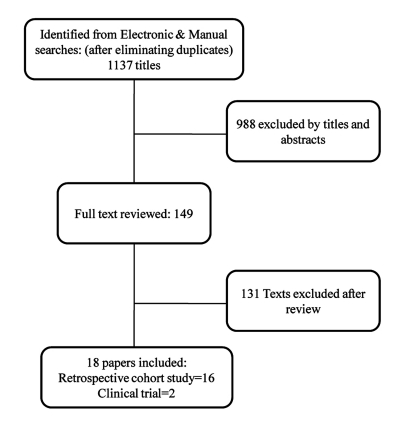
Summary of literature search and study selection

The electronic and manual searches yielded 1137 papers by title and abstract, of which 149 were considered relevant and selected for a full text review. 131 irrelevant reports were excluded (Figure 1). After a full text review, 16 retrospective cohort studies [1][11][12][13][14][15][16][17][18][19][20][21][22][23][24][25], and 2 clinical trials [26][27] were selected for our review (Table 1). Sixteen studies were related to patient survival, and 12 examined graft survival. Study characteristics are summarized in Table 1

**Table 1:** Study characteristics

**Authors**	**country**	**Number of all patients/HCV+ RTRs**	**Patient/graft survival reports **(Yes/No)	**Type of Study**
**Einollahi et al**. (2003) [11]	Iran	1006/45	Yes/yes	Retrospective cohort
**Luan et al. **(2008) [1]	U.S	79337/3708	Yes/no	Retrospective cohort
**Aroldi et al. **(2005) [12]	Italy	541/209	Yes/yes	Retrospective cohort
**Legendre et al. **(1998) [13]	France	499/112	Yes/no	Retrospective cohort
**Gentil et al. **(1999) [14]	Spain	320/85	Yes/yes	Retrospective cohort
**Lee et al. **(2001) [15]	Taiwan	477/136	Yes/yes	Retrospective cohort
**Breitenfeldt et al. **(2002) [16]	Germany	927/123	Yes/yes	Retrospective cohort
**Bruchfeld et al.** (2004) [17]	Sweden	571/51	Yes/no	Retrospective cohort
**Morales et al. **(2004) [18]	Spain	3365/488	Yes/yes	Retrospective cohort
**Ingsathit et al.** (2007) [19]	Thailand	346/22	Yes/no	Retrospective cohort
**Batty et al. **(2001) [20]	U.S	28692/1624	Yes/no	Retrospective cohort
**Mahmoud et al.** (2004) [21]	Egypt	133/80	Yes/yes	Retrospective cohort
**Lin et al. **(2004) [22]	Taiwan	299/129	Yes/yes	Retrospective cohort
**Ridruejo et al. **(2007) [23]	Argentina	396/155	Yes/yes	Retrospective cohort
**Gentil Govantes et al.** (2009) [24]	Spain	5693/1053	No/yes	Retrospective cohort
**Mitwalli et al.** (2006) [25]	Saudi Arabia	448/286	No/yes	Retrospective cohort
**Pereira et al. **(1995) [26]	U.S	75/19	Yes/yes	Clinical trial
**Pereira et al. **(1998) [27]	U.S	103/23	Yes/yes	Clinical trial

### Criteria for inclusion

Two independent reviewers assessed with a standard method each included trial about adult kidney transplant recipients with HCV infection, defined astesting positive for anti-HCV or HCV RNA by polymerase chain reaction (PCR) in serum at the time of enrollment. Also participants were evaluated with regard to patient and kidney outcomes, which were defined as liver-related death and return to dialysis due to HCV infection. Discrepancies were resolved in conference. Other criteria for inclusion were controlled trials and cohort studies that reported patient and graft survival among HCV-infected RTRs. Table 1 shows the characteristics of the studies in this review. Studies that included HCV-infected donors were excluded. Between the trials included in our meta-analysis, there are a few differences in patients and graft outcome (Table 2). Thus, we decided to pool these data for evaluation.

**Table 2:** Follow up and adjusting variables of included articles

**Authors**	**year**	**Adjusted variables**
**Einollahi et al.** (2003) [11]	1995-2001	Donor characteristic (age, source, gender, blood group) and recipient characteristic (age, gender, ESRD etiology, history of diabetes, blood group)
**Luan et al. **(2008) [1]	1995-2004	Recipient characteristics (age, sex, race, diabetes, renal diagnosis,time on dialysis, panel reactive antibody level, availability of private insurance) and donor characteristics (age, living donor, extended criteria donor, cold ischemia time, presence of hypertension, creatinine level, and cause of death)
**Aroldi et al. **(2005) [12]	1972-1989	age
**Gentil et al. **(1999) [14]	1986-1997	Donor characteristic (age, gender, time on dialysis, ESRD etiology, number of transplant, pre-transplant transfusion, peak and immediate pre-transplant immunization, number of HLA A+B and HLA DR mismatches, years of transplant, cold ischemia time, anti HCV Ab, pre-transplant clinical liver disease
**Lee et al.** (2001) [15]	1984-1999	Sex, mode of dialysis, duration of dialysis, diabetes, hypertension, HBV infection, HCV infection, liver function impairment, hepatoma
**Breitenfeldt et al.** (2002) [16]	1978-1994	HBeAg, HCV infection after transplantation, con-comitant HBV and HCV infection, occurrence of acute rejection, age at transplantation and time on dialysis, HBeAg positivity, HBsAg positivity, HCV infection after transplantation, age at transplantation and occurrence of acute rejection.
**Bruchfeld et al. **(2004) [17]	1989-1997	age, sex, diabetes, previous transplantations, type of transplant, and time in RRT for death, HCV, diabetes
**Moraleset al.** (2004) [18]	1990-1994	year of transplant, recipient age, Last panel reactive antibodies, acute rejection, triglycerides, Creatinine, proteinurea
**Ingsathit et al.** (2001) [19]	3.7 year	acute rejection episode, recipient age, long duration of dialysis; diabetes mellitus, delayed graft function, and sex mismatch, Creatinine
**Batty et al. **(2001) [20]	1994-1997	age, race, gender, end-stage renal disease due to diabetes, weight, year of transplant, duration of pre-transplant dialysis, previous transplant, donor and recipient age, donor and recipient race, donor and recipient gender, delayed graft function, antibody induction therapy (combined and also analyzed separately for OKT3 and ALG), and allograft rejection
**Mahmoud et al.** (2004) [21]	1993-1995	donor and recipient age and sex, primary cause of ESRD, HLA mismatch, number of transplants, time on dialysis therapy, number of acute rejection episodes, presence of persistent proteinuria, and year of transplantation.
**Lin et al. **(2004) [22]	1981-2000	Recipient age and sex, donor age and sex, anti HCV Ab, chronic hepatitis, pre-transplant diabetes, , pre-transplant hypertension, pre-transplant coronary artery disease, HLA DR mismatch
**Ridruejo et al**. (2007) [23]	1991-2004	Age, anti-HCV, traditional immunosuppression, rejection
**Gentil Govantes et al.** (2009) [24]	1984-1989 1990–1995 1996–2001 2002–2007	sex and age of the recipient, diabetes as ESRD cause, retransplant status, duration of previous RRT, and transplant year, Transplant time period
**Mitwalli et al.** (2006) [25]	1980-2001	age, sex, blood pressure, type of donor, and immunosuppressive medication , type of donor (living related, living unrelated, and cadaver donors), hepatitis status, hepatitis-positive or hepatitis-negative
**Pereira et al.** (1998) [27]	1987-1990	Effects of hepatitis C infection and renal transplantation on survival in end-stage renal disease. The New England Organ Bank Hepatitis C Study Group

### Review questions and endpoints of interest

Our review aimed to answer two specific questions:

1. What is the effect of HCV infection on renal graft survival?

2. What is the effect of HCV infection on renal recipient survival?

All selected studies used the adjusted relative risk (aRR) and 95% CI of all-cause mortality and graft loss in HCV-positive versus -negative RTRs as study endpoints. Cox proportional hazard 5) (we have converted HR to RR with a formula) analysis was usedin all studiesto calculate independent effects of HCV infection on RTR outcomes after adjustments for potentially contributing factors, such as age, gender, follow-up period, type of transplant, diabetes mellitus, post-transplant plasma creatinine, race, duration of dialysis, donor death etiology, and proteinuria.First-generation enzyme-linked immunoadsorbent assay test before 1991, second generation until 1997 and third generation until now were used to detect HCV infection. Further, serum HCVRNA (PCR) was examined in anti-HCV-positive patients for confirmation of HCV infection in 6 studies.

### Statistical analysis

We pooled outcomes (mortality rates, renal allograft failure), which had been expressed as relative risk (RR) with 95% confidence intervals (CI), using STATA 8. The results of each outcome were analyzed for heterogeneity by Q test (the random effects method of Der Simonian-Laird). Funnel plots, Begg&apos;s rank correlation test, and Egger&apos;s regression asymmetry test were used to assess the existence of publication bias. The Forest plot was used to demonstrate the details of pooled analysis. Combined hazard ratios were assessed by sensitivity analysis.

## Results

### Description of Included Trials

The included studies are summarized in Table 1. Follow up duration and adjusted variables for each study shown in table 2 and adjusted relative risk for mortality and graft loss also presented in table 3. A total of 8348 HCV-infected RTRs before or after kidney transplantation were identifiedfrom 123,228 living and deceased RTRs, asreported in 18 studies. Pereira BJ et al. had 2 studiesin different years. Data on 8 studies (Pereira BJ et al. study 1, Pereira BJ et al. study 2, Legendre C et al., Gentil MA et al., Lee WC et al., Breitenfeldt et al., Bruchfeld et al., and Morales et al.) that were reported before 2005 were also used in a meta-analysis by Fabrizi et al. and Gentil MA et al. confirmed HCV infection detection by immunoblotting, and Bruchfeld (71%), Ridruejo (33.54%), Ingsathit (100%), Mitwalli (100%), and Mahmoud (100%) of hemodialysis patients confirmed it by HCV-RNA (PCR). In response to our request, Einollahi et al. replied that nearly 70% of HCV positive antibody anti HCV antibody positive RTRs were confirmed by HCV RNA (PCR).

**Table 3:** Adjusted relative risk for mortality and graft loss

**Author**	**95% confidence interval**	
	**aRR for mortality**	**aRR for graft loss**
**Einollahi et al.** (2003) [11]	4.308 (2.88-6.4)	2.609 (2.07-3.27)
**Luan et al.** (2008) [1]	1.3 (1.2-1.4)	N.A.^a^
**Aroldi et al.** (2005) [12]	1.65 (1.13-2.42)	1.4 (1.17-1.81)
**Pereira et al. **(1995) [26]	1 (0.49-2.02)	0.95 (0.54-1.67)
**Pereira et al.** (1998) [27]	2.6 (1.15-5.9)	1.3 (0.66-2.58)
**Legendre et al.** (1998) [13]	2.8 (1.4-5.7)	N.A.^a^
**Gentil et al.** (1999) [14]	3.1 (1.2-7.8)	3 (1.8-5)
**Lee et al. **(2001) [15]	1.57 (0.75-1.11)	1.25 (0.75-1.32)
**Breitenfeldt et al.** (2002) [16]	1.93 (1.01-3.42)	N.A.^a^
**Bruchfeld etal.** (2004) [17]	2.23 (1.48-3.34)	1.96 (1.37-2.79)
**Morales et al.** (2004) [18]	1.505 (1.12-2.02)	1.58 (1.27-1.97)
**Ingsathit et al.** (2007) [19]	1.59 (0.28-9.02)	N.A.^a^
**Batty et al.** (2001) [20]	1.23 (1.01-1.49)	N.A.^a^
**Mahmoud et al.** (2004) [21]	0.5 (0.1-1.9)	0.5 (0.3-1.2)
**lin et al.** (2004) [22]	0.3 (0.13-0.65)	0.8 (0.48-1.35)
**Ridruejo et al. **(2007) [23]	1.66 (1.01-2.77)	1.97 (1.18-3.29)
**Gentil Govantes et al.** (2009) [28]	N.A.^a^	1.5 (1.1-1.9)
**Mitwalli et al. **(2006) [25]	N.A.^a^	4.37 (1.8-4.8)

^a^ N.A.:Not Accessible

### Effect on patient and graft survival

**Figure 2:**
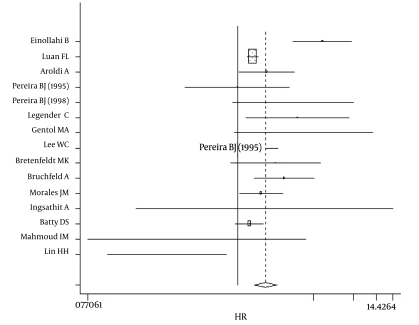
Hazard ratio in HCV-infected recipients for patient survival

**Figure 3:**
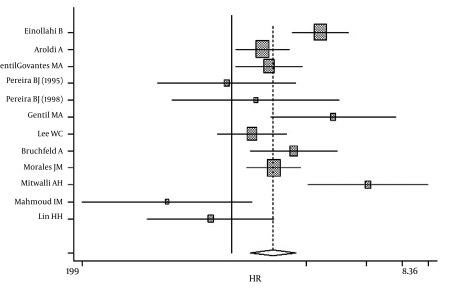
Hazard ratio in HCV-infected recipients for graft survival

The Q-test for heterogeneity revealed p < 0.0001 (Q = 69.81, df = 15) and p < 0.0001 (Q = 66.15, df = 11) for patient and graft survival, respectively. Further, a meta-analysis was done with a random model showed a combined hazard ratio in HCV-infected recipients that was 1.69-fold (1.33-1.97, p < 0.0001) Figure 2 and 1.56 times ( 1.22-2.004, p < 0.0001) (Figure 3) greaterthan in HCV-negative recipients for mortality and graft loss, respectively.

### Publication bias

**Figure 4:**
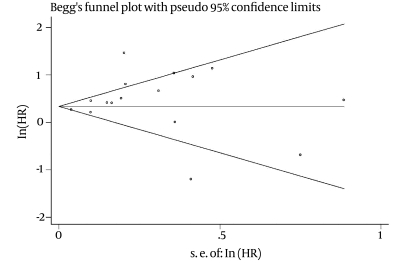
publication bias for patient survival

**Figure 5:**
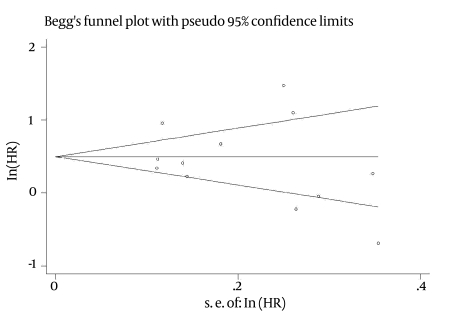
publication bias for graft survival

For patient and graft survival rates, publication bias was examined using Bagg and Manzumdarand Egger's regression asymmetry, both of which were non-significant [(p = 0.753, p = 0.226; Figure 4) and (p = 0.304, p = 0.55; Figure 5, respectively]. Similar results were observed in the funnel plots.

### Sensitivity analysis

**Figure 6:**
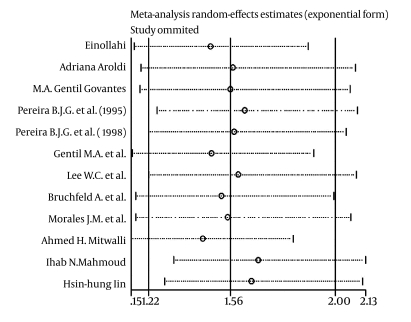
sensitivity analysis for patient's survival

All eligible studies included in meta-analysis. Because the elimination of each study did not have an impact on the combined hazard ratio, the overall estimation was robust Figure 6.

## Discussion

Hepatitis C infection is a risk factor for graft loss and death in renal transplant recipients[8]. Although our report and recent studies have emphasized the detrimental role of hepatitis C in long-term patient and graft survival after renal transplantation[10]several studies have demonstrated that patient and graft survival onHCV infection after renal transplantation arethe same in the shortterm compared withnon-infected renal transplant patients[6]. Conversely, kidney transplantation is a better option for HCV-positive ESRD patients versus remaining on dialysis [1].To better examineHCV-positive RTR outcomes, we performed a meta-analysis using observational studies that used adjusted data of all-cause mortality.

### Impact on patient survival

Consistent withFabrizi's meta-analysis, the aRR for mortality rate in our study was lower than inother studies [4][8][13][14][17], likely due to the greater sample size, early detection, improvement in management, and exact follow-up. Compared with Fabrizi's meta-analysis, which included 8 articles, our study included 18 articles that comprisedmore than 123,000 RTRs, indicatingthat greaterconsideration has been given to the controversy of HCV-infected RTR outcomes and kidney transplantation in the past5 years. Several studies have demonstrated lower patient and graft survival in HCV-positive RTRs, related in part to associated complications, such as cirrhosis, hepatocellular carcinoma, cardiovascular disease, diabetes mellitus, sepsis, higher PRA, and deceased kidney donation [1][10].

### Impact on graft survival

In our study,the aRR for graft loss was similar to that in Fabrizi's meta-analysis. Although during the first 5-10 years, graft and patient survival was apparently similar between negative and positive HCV-infected RTRs [4], HCV-associated glomerulonephritis, proteinuria, and diabetic nephropathy can progressrapidly to chronic allograft nephropathy [6].

### Role of other factors in mortality

It appears that the increased mortality in anti-HCV-positive patients was partially related to mortality dueto causes other than HCV infection. According to a novel risk score for mortality in RTRs [29], the risk score for HCV (1.5) was not more than age above 40 years in comparison to younger than 40 (2.2-6.7), pre-transplant diabetes mellitus (1.8), post-transplant diabetes mellitus (1.5), serum creatinine levels at the first year after transplantation (1.7), and proteinuria greater than 1g during the first year of operation (2.7). In a recent meta-analysis, mortality due to liver complications, such as cirrhosis and hepatocellular carcinoma, among HCV-infected RTRs increased in most studiesthat were included, with anRRof 1.79, compared with HCV-negative recipients [6]. In a systematic review, cardiovascular and infectious diseases were also important causes of death in HCV-positive RTRs [6].

Because mortality and graft loss are multifactorial, we used the aRR that had been obtained by the Cox regression model in each study to appraise the isolated influence of HCV infection on patient and graft survival. In contrast to studies that reported a negative impact, the majority of studies that demonstrated a positive impact of transplantation on HCV-infected patient and graft survival rates did not use the Cox regression model; consequently, studies that observed a positive impact or not on HCV-positive patients were excluded from this systematic review and meta-analysis. Although our study and other similar articles on the effect of HCV infection on patient and graft survival did not have any publication bias, it appears that we included only papers with a negative impact (Figure 2,Figure 3).Consistent with previous surveys, we observed that the aRR of all-cause mortality and graft failure was significantly higher for seropositive HCV recipients after kidney transplantation.

### Role of immunosuppression in HCV-positive kidney transplant recipients

The progression of liver failure in HCV-positive RTRs following immunosuppression is debated. While previous studies have illustrated a detrimental effect on liver function in these patients [10][11], more recent studies have observed relatively slow development of liver fibrosis in such patients [1]. Luan (2008) performed a study using national data and Cox regression analysis to estimate hazard ratios, adjusted for donor, recipient, and transplant variables. A total of 3708 HCV-positive and 75,629 HCV-negative kidney transplant recipients were included, wherein no calcineurin inhibitors (cyclosporine A or tacrolimus) or steroids had a significant impact on patient mortality. Moreover, the use of mycophenolytemofetile (MMF) not only was associated with a significantly reduction in mortality rate, it also had a protective effect [1], despite it sassociation with increased HCV viremia[1]. According to another study, HCV replication increases after kidney transplantation, likely due to immunosuppression [1]. In contrast, in cultured hepatocytes, cyclosporine A, but not tacrolimus, prevents HCV replication. Notably, more than 50% of HCV-positive kidney transplant recipients who are treated with cyclosporine A have stable liver function and decreased liver fibrosis [1]. Nevertheless, in HCV-positive kidney transplant patients, the use of antibody induction has no correlation with viral load [1] and does not have a negative influence on patient survival in these patients [6]. It appears that the anti-HCV activity of cyclosporine A differs from its immunosuppressive effects [10]. Thus, based on the protective effects of new immunosuppressive drugs, such as MMF and cyclosporine, we hope for greater survival of HCV-positive renal transplant recipients. Yet, controversy still exists regarding the impact of HCV infection on the outcomes of renal transplantation.

### Limitations

The majority of articles are not complete; some did not consider Cox regression, and the aRR for patient and graft survival was not reported. Some contributing factors, such as alcohol or drug consumption, were not noted. After renal transplantation, HCV-positive patients have lower patient and graft survival rates compared with HCV-negative patients. However, HCV infection is not a contraindication for renal transplantation; and HCV therapy before transplantation is important to improve the outcome of the patients after transplantation.
